# Treatment of Intrabony Defects with a Combination of Hyaluronic Acid and Deproteinized Porcine Bone Mineral

**DOI:** 10.3390/ma14226795

**Published:** 2021-11-11

**Authors:** Darko Božić, Ivan Ćatović, Ana Badovinac, Larisa Musić, Matej Par, Anton Sculean

**Affiliations:** 1 Department of Periodontology, School of Dental Medicine, University of Zagreb, HR-10000 Zagreb, Croatia; badovinac@sfzg.hr (A.B.); lmusic@sfzg.hr (L.M.); 2 Private Dental Practice, HR-52100 Pula, Croatia; ivan.catovic@gmail.com; 3 Department of Endodontics and Restorative Dentistry, School of Dental Medicine, University of Zagreb, HR-10000 Zagreb, Croatia; mpar@sfzg.hr; 4 Department of Periodontology, School of Dental Medicine, University of Bern, CH-3010 Bern, Switzerland; anton.sculean@zmk.unibe.ch

**Keywords:** hyaluronic acid, periodontal regeneration, intrabony defects, microsurgery, periodontitis, surgical flaps

## Abstract

Background: this study evaluates the clinical outcomes of a novel approach in treating deep intrabony defects utilizing papilla preservation techniques with a combination of hyaluronic acid (HA) and deproteinized porcine bone mineral. Methods: 23 patients with 27 intrabony defects were treated with a combination of HA and deproteinized porcine bone mineral. Clinical attachment level (CAL), pocket probing depth (PPD), gingival recession (REC) were recorded at baseline and 6 months after the surgery. Results: At 6 months, there was a significant CAL gain of 3.65 ± 1.67 mm (*p* < 0.001) with a PPD reduction of 4.54 ± 1.65 mm (*p* < 0.001), which was associated with an increase in gingival recession (0.89 ± 0.59 mm, *p* < 0.001). The percentage of pocket resolution based on a PPD ≤4 mm was 92.6% and the failure rate based on a PPD of 5 mm was 7.4%. Conclusions: the present findings indicate that applying a combined HA and xenograft approach in deep intrabony defects provides clinically relevant CAL gains and PPD reductions compared to baseline values and is a valid new approach in treating intrabony defects.

## 1. Introduction

Periodontitis is a non-communicable chronic inflammatory disease caused by periodontal pathogenic biofilm [1,2,3,4] and with 743 million affected people worldwide in its severe form, it is the sixth most prevalent disease globally [5,6]. If left untreated, the disease leads to tooth loss and has a significant social and economic impact [7].

Following non-surgical therapy, it is common that some deep intrabony defects remain, presenting an increased risk of disease progression with further attachment loss that could lead to tooth loss [8,9].

Therefore, one of the ultimate goals in periodontology is to achieve periodontal regeneration and change the prognosis of questionable or hopeless teeth to maintainable. Over the last several decades, periodontal regenerative procedures have seen a change in flap designs and materials used to promote periodontal regeneration [10]. Human histological studies have shown that different materials can promote periodontal regeneration to various success [11]. In addition, several clinical studies have shown long-term stability of clinical attachment gain of periodontally compromised teeth when treated with regenerative procedures benefitting the patient in retaining their teeth [12,13,14,15,16]. 

Results from systematic reviews evaluating the clinical outcomes obtained with various biologics/growth factors for regenerative periodontal therapy have shown positive clinical effects evidenced by the gain of clinical attachment and pocket-depth reduction [17,18]. Based on the accumulating evidence on the benefits of periodontal regenerative procedures and the current evidence related to the use of these procedures, very recently Nibali pointed out that periodontal regenerative procedures should be the treatment of choice for intrabony defects [19].

Over the last several years, another emerging molecule serving as a potential candidate for periodontal regeneration is the hyaluronic acid (HA), a key extracellular matrix component involved in cell migration. As a major component of the extracellular matrix, it is expressed in various cells of the periodontium [20]. Furthermore, its main receptor, CD44, a cell surface molecule, is expressed by PDL cells and cementoblasts [21,22,23,24]. Of importance for periodontal regeneration, it has been shown that the interaction between CD44-HA in PDL cells is critical for the proliferation and migration of these cells [23,24]. Furthermore, data from other in vitro studies revealed that HA induces early osteogenic differentiation of PDL cells [25], increases the migratory and proliferative properties of gingival fibroblasts [26] and maintains the stemness of mesenchymal stromal cells and pre-osteoblasts, making it a valid candidate for bone and periodontal regeneration [27]. In addition, HA can induce proliferation and osteogenic differentiation of bone marrow stromal cells and pre-osteoblastic cells, having an important role in the early and late stages of bone formation [28,29]. Recent animal studies have shown an increase in bone formation and improved wound healing after applying HA in chronic pathology-type extraction sockets, comparable to that observed following the use of BMP-2 [30,31]. These findings are in line with those from two animal studies evaluating the effects of HA in the treatment of intrabony and recession defects revealing periodontal regeneration evidenced through the formation of cementum, periodontal ligament and bone [32,33]. 

Thus, taken together, the current evidence suggests that HA may not only positively influence periodontal regeneration but may also have a potential role in bone formation. Indeed, recent systematic reviews have shown that the adjunctive use of HA in both non-surgical and surgical periodontal therapy may have a positive effect on the clinical outcomes evidenced by pocket probing depth (PPD) reduction, clinical attachment level (CAL) gain and reduction of bleeding on probing (BOP). However, the authors also suggested a need for further studies that would investigate HA in different clinical scenarios [34,35].

A recent randomized clinical study with a 24-month follow-up provided further evidence of a positive effect HA has on CAL gains when enamel matrix derivative (EMD) was compared with HA in the surgical treatment of intrabony defects. The study showed that both materials may lead to comparable clinical results in terms of PPD reductions and CAL gains [36]. To further improve the clinical outcomes of regenerative therapy by stabilizing the blood clot and providing space for periodontal regeneration, bone replacement grafts were combined with an enamel matrix derivative (EMD). The combination of EMD and bone replacement grafts resulted in an additional gain of CAL (i.e., 1mm) when compared to the use of EMD alone [37]. Interestingly, when HA was used alone or combined with a volume stable collagen matrix, the combination approach yielded a higher but not statistically significant amount of new cementum, new periodontal ligament and new bone compared to the use of HA alone [33]. These very recent findings suggest that a filler material might be beneficial for periodontal regeneration when using HA, thus stabilizing the blood clot and providing flap support to prevent its collapse into the intrabony defect and creating more space for periodontal regeneration.

However, no clinical data are currently available evaluating the combined use of HA with a bone replacement graft in intrabony defects. Therefore, this pilot case series aimed to clinically evaluate, for the first time, the healing of intrabony defects treated with a combination of HA and a bone replacement material.

## 2. Materials and Methods

### 2.1. Study Setting and Ethical Considerations

The study was conducted at the Department of Periodontology, School of Dental Medicine, University of Zagreb, between June 2019 and December 2020. All clinical procedures were performed in full accordance with the Declaration of Helsinki and the Good Clinical Practice Guidelines. Each patient provided written informed consent. The study protocol was approved by the Ethical committee of the School of Dental Medicine University of Zagreb, Croatia (05-PA-30-XXI).

### 2.2. Study Population

Patients referred to the Department of Periodontology for periodontal treatment were consecutively screened for study inclusion. Before enrolling, all patients underwent cause-related therapy, consisting of oral hygiene instructions and scaling and root planing with machine-driven and hand instruments. Splinting of mobile teeth was performed if necessary.

The inclusion criteria were as follows: (i) diagnosis of stage III or IV periodontitis; (ii) good general health with no systemic diseases that could contraindicate surgery, no medications that could affect the periodontal status, uncontrolled or poorly controlled diabetes, no pregnancy or lactation; (iii) patients had to have at least one intrabony defect with PPD ≥ 6 mm, CAL ≥ 6 mm and an intrabony component ≥ 4 mm measured on digital periapical radiographs that predominantly involved the interproximal area of the affected tooth; (iv) FMPS and FMBS ≤ 20% following non-surgical treatment [38]; (v) vital teeth or teeth with properly performed endodontic treatment.

Exclusion criteria were: (i) teeth with degree III mobility, furcation involvement, or inadequate endodontic treatment and/or restoration; (ii) heavy smokers (more than 10 cig/day). We enrolled 23 systemically healthy patients in this case-series study (16 females and 7 males, mean age 54.59 ± 10.24, age range: 35–85 years), 4 of whom were smokers (<10 cigarettes per day).

### 2.3. Surgical Procedures

All surgical procedures were performed by an experienced periodontist (D.B.) with more than 20 years of clinical experience. Following local anesthesia, the defect-associated papillary area was either accessed with the simplified papilla preservation flap (SPPF) [39] or with the modified papilla preservation flap (MPPT) [40]. The flap design was chosen based on the width of the interdental space; when 2 mm or less SPPF was utilized, while when the interdental space was wider than 2 mm MPPT was used.

The incisions were intrasulcular in order to preserve the width and the height of the defect-associated papilla. The mesial-distal extension was at least one tooth mesial and distal to the defect site in order to provide access to the base of the bony defect and allow proper visualization and debridement of the defect. Periosteal incisions were never performed.

Following a full-thickness flap reflection, granulation tissue was removed from the intrabony defect using curettes and microscissors. Scaling and root planing of the root surface was performed with both hand curettes (Gracey, Hu-Friedy, Chicago, IL, USA) and power-driven instruments (SONICflex LUX, KaVo Dental GmbH, Biberach, Germany).

### 2.4. Application of the Hyaluronic Acid (HA) and Xenograft

**Figure 1:**
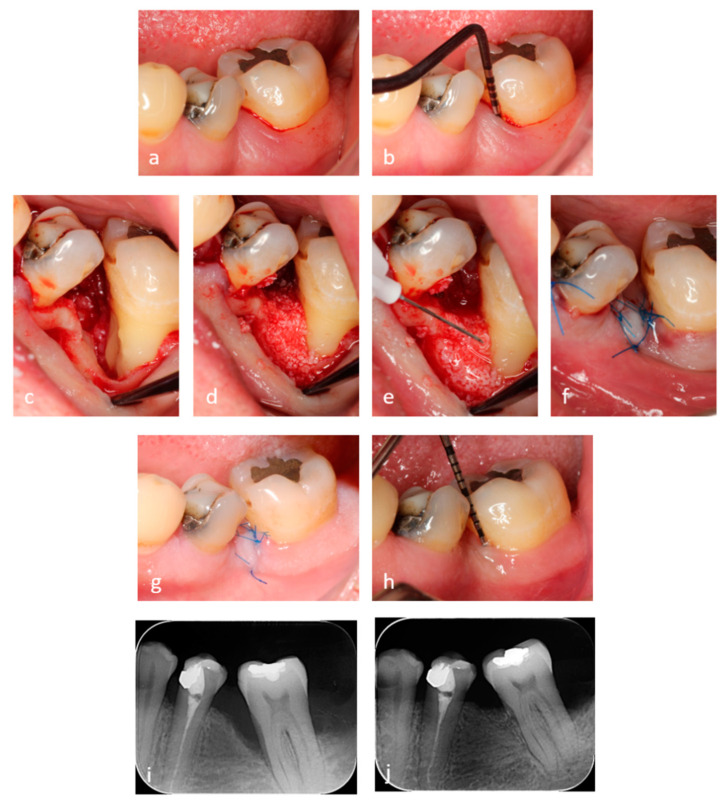
(**a**,**b**) Preoperative view and a pocket probing depth (PPD) of 9 mm. (**c**) Clinical view of the 1-wall intrabony defect and the instrumented root surface with a thin layer of hyaluronic acid (HA) applied. (**d**) Mixture of HA and xenograft gently packed in the intrabony defect. (**e**) HA layer applied before suturing. (**f**) Passive primary wound closure. (**g**) 14 days healing before suture removal. (**h**) PPD of 3 mm at 6 months. (**i**) Pre-operative radiographic finding. (**j**) Radiographic finding at 6 months.

**Figure 2:**
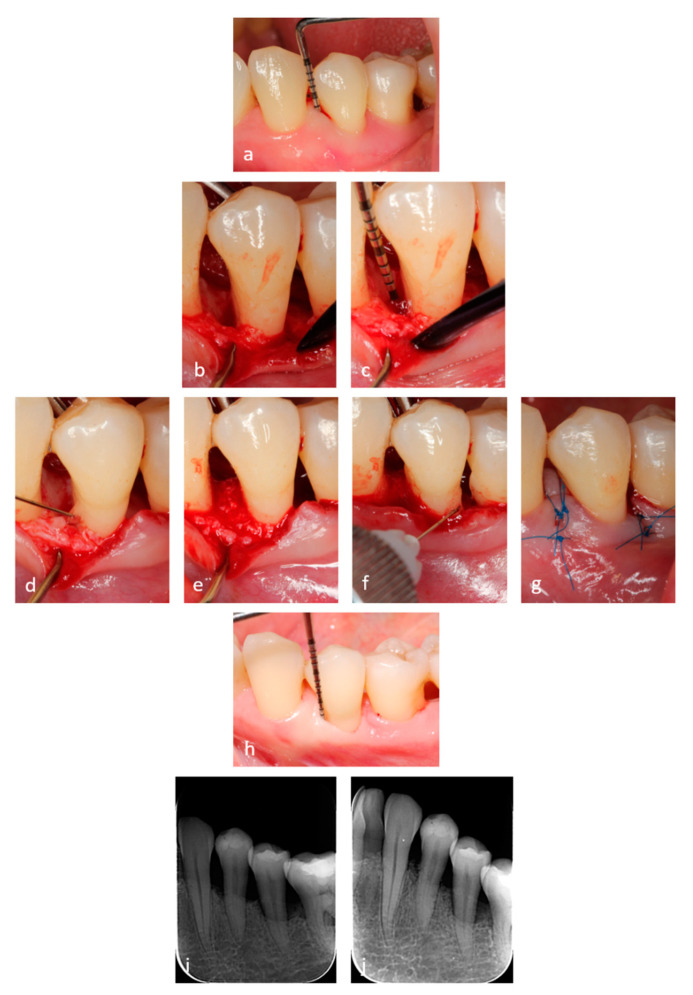
(**a**) Preoperative view and PPD depth of 8 mm. (**b**,**c**) Clinical intraoperative view of the defect with a 7mm depth of the intrabony component. (**d**) Clinical view of the instrumented root surface with a thin layer of HA applied. (**e**) Mixture of HA and xenograft gently packed in the intrabony defect. (**f**) HA layer applied before suturing on the surgical area. (**g**) Passive primary wound closure. (**h**) PPD of 2 mm at 6 months. (**i**) Pre-operative radiographic finding. (**j**) Radiographic finding at 6 months.

At the end of the instrumentation, the defect was rinsed with sterile saline and EDTA (sterile 24% EDTA gel, pH 6.7; PrefGel, Straumann, Basel, Switzerland) was applied on the instrumented root surface for 2 min. The defect area was then carefully rinsed with saline and a thin layer of cross-linked HA gel (HYADENT BG^®^, BioScience, Germany: a gel formulation containing cross-linked HA (1000 kDA HA monomers) and non-cross-linked HA (2500 kDA) in a ratio 8:1, made from biotechnologically produced synthetic HA) was applied on the root surface. Afterwards a 1:1 ratio mixture of HA and deproteinized porcine bone mineral (THE Graft, Purgo Biologics Inc., Korea) was gently packed into the intrabony defect to the level of the bone, not overfilling the defect. The flaps were repositioned and primary wound closure was achieved with a horizontal internal mattress suture at the base of the papilla and a single interrupted suture to connect the tips of the papillae. The papillae were sutured using the monofilament non-resorbable 5-0/6-0 suturing material (Ethilon‚ Ethicon, Johnson and Johnson, Somerville, NJ, USA) (Figure 1 and Figure 2).

### 2.5. Post-Surgical Instructions and Plaque Control

Patients received systemic antibiotic therapy amoxicillin + clavulanic acid 1 g/day for seven days. Pain control was obtained by 400 mg ibuprofen three times per day for the first 24 h and subsequently based on the patient’s need. Each patient was advised to rinse twice per day with a 0.2% chlorhexidine gluconate solution (Parodontax extra, GSK, Brentford, UK) for 4 weeks. Smoking patients were asked to refrain from smoking during the first 4 post-operative weeks. Sutures were removed 14 days following surgery, and the patients were instructed to brush with a post-surgical soft toothbrush. The use of a soft toothbrush was discontinued after 3 months when a medium bristled toothbrush was re-introduced. Each patient received professional tooth cleaning during the monthly control appointments for the following 6 months.

### 2.6. Outcome Measures

The primary outcome measure defined in the study was the change in CAL between baseline and 6 months. The secondary outcomes were changes in PPD and REC.

All clinical measurements were carried out by a single examiner (I.Ć.) at baseline and 6 months after surgery. Prior to the study, the examiner was calibrated to reduce the intraexaminer error (=0.893) in order to reach reliability and consistency. Pocket probing depths and gingival recessions were rounded to the nearest 0.5 mm at the deepest part of the interproximal site. Clinical attachment level was calculated as a sum of PPD and REC. For the first month after the surgery, the primary closure of the surgical sites was evaluated on a weekly basis.

### 2.7. Statistical Analysis

The assumption of normality of distribution was evaluated using Shapiro-Wilk’s test and the inspection of normal Q-Q plots. Homogeneity of variances was verified using Levene’s test. For PPD, CAL, and REC, baseline values were compared to the values measured after 6 months using paired *t*-tests. The relationships for binary combinations of outcome variables were explored using Pearson’s correlation analysis, except for the variable EHI, which was analyzed using Spearman’s correlation due to the violation of normality. *p*-values lower than 0.05 were considered statistically significant. The statistical analysis was performed using SPSS (version 25, IBM, Armonk, NY, USA).

## 3. Results

### 3.1. Baseline Data

A total of 27 defects were treated with 17 intrabony defects located in the maxilla, and 10 were in the mandible (8 incisors, 3 canines, 9 premolars, 7 molars).

The mean distance from the cementoenamel junction (CEJ) to the bottom of the defect (CEJ-BD) was 10.54 ± 3.23 mm, and the mean intrabony component (IB) was 7.24 ± 2.46 mm. The mean intrabony width of the osseous defect (IBW) was 3.44 ± 0.96, and the mean value of the radiographic defect angle was 26.4 ± 8.43°. From 27 intrabony defects, 13 were 2-wall, 7 were 1-wall, 6 were 3-wall and one was a crater defect. Patient and defect characteristics are presented in Table 1.

### 3.2. Early Wound Healing

Primary closure of the incision lines was achieved in 100% of the cases. We assessed the early wound healing index (EHI) during wound healing [41]. At the 2-week suture removal time of the 27 treated sites, 13 sites had an EHI score of 1, 10 sites had EHI 2, 3 sites had an EHI score of 4, and 1 site had a score of 3. Spearman’s correlation analysis found no significant relationship between EHI score and CAL gains and PPD reductions.

### 3.3. Clinical Outcomes at 6 Months

Six months after the surgery, the results showed statistically significant changes for PPD, CAL and REC (*p* < 0.001). The mean residual PPD after 6 months was 3.35 ± 0.72 mm with a decrease of 4.54 ± 1.65 mm. Of the 27 treated sites 14 sites had a residual PPD of 2–3 mm, 11 sites had 4 mm and 2 sites had a PPD of 5 mm. The CAL also significantly changed from baseline to 6 months with an average CAL gain of 3.65 ± 1.67 mm. Twelve sites showed CAL gains of 2–3 mm, and 3 sites had 4mm of CAL gain and 11 sites reached CAL gains of ≥5 mm. At 6 months, there was a mean increase of 0.89 ± 0.59 mm in REC compared with the baseline value (0.83 ± 0.67 mm). Clinical baseline data and outcomes 6 months after treatment, and the frequency distribution of CAL gains, residual PPD and REC after 6 months are shown in Table 2 and Table 3.

A separately conducted statistical analysis was done for the four smoking patients for CAL gains, PPD reductions and REC changes and no statistical significance was found compared to the non-smoking patients.

Correlations among interval variables were analyzed and CAL gain significantly and positively correlated with preoperative PPD and CAL (respectively: r = 0.812, *p* < 0.001, r = 0.731, *p* < 0.001), PPD reduction (r = 0.936, *p* < 0.001), IB component (r = 0.494, *p* < 0.009) and CEJ-BD (r = 0.526, *p* < 0.005).

Similar correlations were found for PPD reduction, where PPD reduction strongly correlated with preoperative PPD and CAL (respectively: r = 0.903, *p* < 0.001, r = 0.815, *p* < 0.001), CAL gain (r = 0.936, *p* < 0.001), IB component (r = 0.484, *p* < 0.01) and CEJ-BD (r = 0.601, *p* < 0.001).

**Figure 3:**
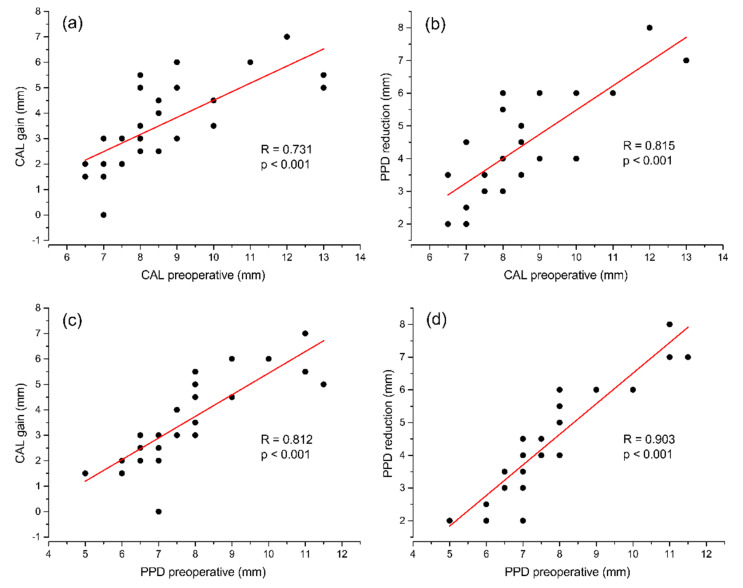
Correlations among interval variables. (**a**) CAL gain compared to preoperative CAL. (**b**) PPD reduction compared to preoperative CAL. (**c**) CAL gain compared to preoperative PPD. (**d**) PPD reduction compared to preoperative PPD.

The correlations with the highest Pearson’s coefficient are shown in Figure 3.

## 4. Discussion

This case-series study shows that the combination of HA and a xenograft can yield substantial clinical improvements in deep interdental intrabony defects. Utilizing well-established surgical techniques to obtain primary closure and wound stabilization, the results of this study appear to point to the potential relevance of HA to achieve clinically relevant improvements in terms of CAL gains and PPD reductions in deep intrabony defects.

HA is a molecule that has been reported to stimulate the proliferation of gingival fibroblasts, PDL cells, induce osteogenic differentiation of bone marrow stromal cells (BMSC) and is well known for promoting angiogenesis and neovascularization [26,29,42]. Recent histological animal studies that evaluated HA for periodontal regeneration confirmed that the application of HA can promote periodontal regeneration in 2-wall and dehiscence defects [32,33], possibly by interacting with CD44 during the early phases of periodontal tissue regeneration [43].

In recent decades’ various biological agents, either alone or combined with bone grafts, have gained much interest in regenerative periodontal surgery, and clinical trials have shown their efficacy in achieving significant CAL gains and PPD reductions [17,44,45,46]. All studies have shown that when biologics are combined with bone grafts greater CAL gains and PPD reductions are achieved, further improving clinical outcomes.

Recently HA has gained attraction with several clinical trials conducted to assess its efficacy in periodontal regeneration [36,47,48]. Of particular interest to our results is the study by Pilloni since they used the same hyaluronic acid as we did. In their randomized clinical trial comparing EMD to HA over 24 months, they achieved CAL gains of 2.19 ± 1.11 mm in the HA group and 2.94 ± 1.12 mm in the EMD group and PD reductions of 4.5 ± 0.97 mm for EMD and 3.31 ± 0.70 mm for HA.

Comparing the present results to those mentioned above, it appears that the use of HA combined with a bone grafting material may further improve the clinical outcomes compared to the use of HA alone (i.e., CAL gains of 3.65 ± 1.67 mm vs. 2.19 ± 1.11 mm, and PD reductions of 4.54 ± 1.65 mm vs. 3.31 ± 0.70 mm). One of the reasons for these observed differences could be due to the fact that HA, similar to other biologics, has a fluid consistency which then prevents it to possess a sufficient space-making potential needed for periodontal regeneration. This could then lead to the collapse of the mucoperiosteal flap and subsequently limit the outcomes of regenerative surgery. By combining a bone-grafting material with HA this could then provide sufficient support to the mucoperiosteal flap preventing its collapse into the defect, thus stabilizing the blood clot and providing additional space needed for periodontal regeneration. These results are in line with those of a recent systematic review that analyzed the application of EMD with and without bone grafts (46). When EMD was combined with bone grafts the CAL gains were 3.76 ± 1.07 mm compared to 3.32 ± 1.04 mm when EMD was used alone while the PPD reductions were 4.22 ± 1.20 mm in the combination group compared to 4.12 ± 1.07 mm when EMD was alone. However, the increase in REC amounted 0.76 ± 0.42 mm, which is comparable to our value of 0.89 ± 0.59 mm. The reason for such an increase in the recession in our study could be due to the fact that the majority of our defects were 1- and 2-wall or combinations of the two with little support of the surrounding tissues. This assumption is in line with those of a previous study by De Leonardis and Paolantonio where they reported CAL gains of 3.47 ± 0.65mm and PPD reductions of 4.00 ± 0.42 mm following the combination of EMD with a bone graft in 1- and 2-wall defects [49]. The importance of intrabony defect morphology, number of walls, radiographic angle and depth of the intrabony component was recently analyzed in a systematic review and meta-analysis [50]. The authors concluded that these parameters affect the clinical outcomes, indicating that deeper, narrower defects and defects with more walls are associated with improved clinical and radiographic outcomes. To overcome the issue of a negative effect of a defect morphology on the clinical outcome, the additional use of filler material is recommended in these non-space maintaining defects to increase the stability of the blood clot and create space for the regeneration process. In this context, the present study results in terms of CAL gains and PPD reductions are comparable to those of other studies that have utilized different growth factors combined with bone grafting materials, thus supporting the use of combination approaches for periodontal regeneration [17,44,45,46].

However, CAL gain changes alone have to be considered carefully, especially if periodontal pockets deeper than 4mm still remain since they may represent a significant risk factor for long-term disease progression/recurrence [9]. Therefore, in a recent publication, Trombelli et al. [51] proposed a composite outcome of combined CAL gains and PPD ≤ 4 mm examining the frequency of these clinically relevant outcomes. Furthermore, in a recent systematic review by Aimetti et al. the authors assessed the frequency of reported pocket resolution (PPD ≤ 3 mm and PPD ≤ 4 mm) outcomes in the regenerative treatment of intrabony defects utilizing papilla preservations techniques (PPTs) [52]. Their final analysis included 12 randomized clinical trials and found that the pocket resolution with a PPD ≤ 3 mm was achieved in 61.4% and with a PPD ≤ 4 mm in 92.1%. However, for the PPD ≤ 3 mm, the results ranged from 28.6% to 93.3%, while for PPD ≤ 4 mm, the range was less variable, ranging from 71.4% to 100%. In the present study, we found that in 51.9% of the sites we had a remaining PPD ≤ 3 mm, and in another 40.7% of the sites a PPD of 4 mm, with two sites having a PPD of 5 mm, 7.4%. However, when we consider PPD of 4mm as the cut-off value, the overall percentage of pocket resolution is 92.6%, which is in line with the aforementioned systematic review. When we compare the 6 months results from Trombelli et al. [51] to our 6 months’ results, similarities in terms of CAL gains, pocket closure percentages and failure rates, defined as PPD > 4 mm, are observed. CAL gains in the Trombelli study were 3.7 mm, compared to our 3.65 mm, the average residual PPDs were 3.7 mm compared to our 3.35 mm, and failure rates indicated by a PPD > 4 mm were 7% compared to our 7.4%. When we look at pocket closure percentages, the authors reported 79.6% compared to 92.6% in our study. This discrepancy could be due to the bigger number of treated sites in their study n = 103, compared to *n* = 27 in our study. However, these results show that the combined approach using HA + xenograft with PPTs is able to achieve clinically relevant PPD reductions, CAL gains and pocket resolution compared to other clinical studies.

Limitations of the present study are the lack of a control group treated with HA alone and the short-term follow-up of 6 months, although there are a number of multicenter clinical trials with 6 months of follow-up [44,45,51]. Furthermore, although radiographs were taken, they were not standardized and, therefore, no precise hard tissue measurements were possible.

## 5. Conclusions

In conclusion, the application of a combined approach of HA and a xenograft in deep intrabony defects provides clinically relevant CAL gains and PPD reductions with over 90% of pocket closure based on a PPD of 4 mm. It can thus be anticipated that this approach may represent a new treatment option for deep intrabony defects. Therefore, randomized clinical trials comparing the use of HA alone with HA plus bone graft are warranted to shed light on the clinical relevance of this novel treatment approach.

## Figures and Tables

**Table 1:** Patient and defect characteristics (respectively, *n* = 23 and *n* = 27).

**Study population**	
Age (years, mean ± SD)	54.59 ± 10.24
Gender (male/female)	7/16
Smoking (yes/no)	4/19
**Defect characteristics**	
Dental arch (maxillary/mandibular)	17/10
Tooth type (incisor/canine/premolar/molar)	8/3/9/7
CEJ-defect bottom (mean ± SD, mm)	10.54 ± 3.23
Intrabony component (mean ± SD, mm)	7.24 ± 2.46
Intrabony width (mean ± SD, mm)	3.44 ± 0.96
X-ray angle (mean ± SD, degree)	26.4 ± 8.43
**Defect configuration**	
1-wall	7
2-wall	13
3-wall	6
Crater	1

CEJ, cementoenamel junction; SD, standard deviation.

**Table 2:** Clinical outcomes at baseline and 6 months after treatment (*n* = 27).

Variable	Baseline	6 Months	Change	Significance, *p* ^a^
PPD (mm)	7.89 ± 1.60	3.35 ± 0.72	4.54 ± 1.65	<0.001
CAL (mm)	8.72 ± 1.82	5.07 ± 1.28	3.65 ± 1.67	<0.001
REC (mm)	0.83 ± 0.67	1.72 ± 0.90	0.89 ± 0.59	<0.001

^a^ Paired *t*-test. PPD, pocket probing depth; CAL, clinical attachment level; REC, gingival recession.

**Table 3:** Frequency distribution of CAL gains and residual PPD after 6 months.

	CAL Gain		Residual PPD	
	*n*	%	*n*	%
0–1 mm	1	3.7	0	0
2–3 mm	12	44.4	14	51.9
4 mm	3	11.1	11	40.7
5 mm	6	22.2	2	7.4
≥6 mm	5	18.5	0	0

CAL, clinical attachment level; PPD, pocket probing depth.

## Data Availability

The data presented in this study are available on request from the corresponding author.
